# Supplementary material for: An improved harris hawks optimization algorithm based on chaotic sequence and opposite elite learning mechanism
Source: PLoS One. 2023 Feb 22;18(2):e0281636. doi: 10.1371/journal.pone.0281636 (PMC9946268; doi:10.1371/journal.pone.0281636)
Supplement: Attachment — Submitted filename: Response to Reviewers.docx [file pone.0281636.s001.docx]

These are my responses to each question. Thank you for your advice in your busy schedule, I thought about each question carefully and gave an explanation. Due to my limited research skills, there may be some incorrect answers, please criticize and correct me. Thanks again! Best wishes to you!

**Reviewer #1:**

1. Please complete the Abstract within 200 words and it should be very precise.

Reply: It has been modified, now there is only 181 words.

2. Motivations of the paper are not clear.

Reply：The HHO still has some limitations, such as the multiplicity of solutions generated by a randomized policy that is finite in the initialization phase. Moreover, because global exploration is only performed in the first half of the iteration, it is difficult to balance the global exploration and local exploitation capacities by using the escaping energy of prey, so the algorithm may converge slowly, has low solution accuracy and prematurely falls into a local optimal solution. To conquer the limitations of HHO, an improved HHO algorithm has been proposed in this paper.
3. Disadvantages of the existing schemes must be discussed. It is important to justify and motivate the new work.

Reply：HHO is a new swarm intelligence optimization algorithm proposed by Heidari et al.^[9]^ in 2019 that mimics the way Harris eagles find and chase prey in nature, including global exploration, local besiege and pounce behaviour. However, similar to the other SI algorithms, the HHO still has some limitations, such as the multiplicity of solutions generated by a randomized policy that is finite in the initialization phase. Moreover, because global exploration is only performed in the first half of the iteration, it is difficult to balance the global exploration and local exploitation capacities by using the escaping energy of prey, so the algorithm may converge slowly, have low solution accuracy and prematurely fall into a local optimal solution.

4. In the Introduction, there must be point-wise contributions.

Reply：the contributions of this paper are as follows:(i) a chaotic sequence chaotic sequence recombination mechanism (CSRM) strategy is proposed, which enhances the distribution of the initialized solutions in the search space, and accelerates the convergence rate of HHO;(ii) the generalized opposition-based learning recombination mechanism (OBLRM) is proposed, which can have the opportunity to carry out global search in the later period of iteration to jump out of the local optimum and improve the accuracy of the solution.

5. The authors have not given Related Works section, so the Introduction section must be very strong.

Reply：In the introduction section, firstly, the development and application of optimization algorithm are described, secondly, the research situation of HHO algorithm and many HHO variants are summarized, finally, the shortcomings of HHO algorithm are summarized and the improved methods and contributions of this paper are proposed.  Related work is integrated into the introduction section, and 47 references have been cited in this paper.
6. Section 2 must be renamed as “Background Studies”.

Reply：it has been modified
7. What are the major advantages of Harris hawk optimization? Mention it.

Reply: HHO is a new swarm intelligence optimization algorithm proposed by Heidari et al.^[9]^ in 2019 that mimics the way Harris eagles find and chase prey in nature, **which has strong global search capability and adjusts few parameters**. It has been modified.

8. In Eq. (3), how the value of T is changed?

Reply: T denotes the maximum number of iterations, which is defined by experience.
9. Rename section 3 as “Proposed Scheme”. Moreover, motivations must be mentioned.

Reply: it has been modified in section 3. To improve the diversity of initial population and enhance the ability to jump out of local optimal solution of HHO algorithm, the specific implementation of the proposed algorithm is described in detail in this section. Two recombination mechanisms, the chaotic sequence recombination mechanism (CSRM) and generalized opposition-based learning recombination mechanism (OBLRM), are introduced to enhance the performance of the HHO algorithm. The improved HHO algorithm does not change the structure of the HHO. Fig. 3 shows the optimization process of the proposed algorithm.

10. What are the used of Opposite Elite Learning Recombination Mechanism?

Reply: It is well known that in the HHO algorithm, even if the selected region is not globally optimal, the global search is no longer carried out in the later periods of iteration, so the HHO tends to converge to the local optimum prematurely. However, when the opposite elite learning recombination mechanism is embedded in the HHO algorithm, the improved algorithm can have the opportunity to carry out global search in the later period of iteration to jump out of the local optimum and improve the accuracy of the solution. It is shown in section 3.2.

11. It is difficult to identify the novelty of the proposed scheme.

Reply: HHO is a new swarm intelligence optimization algorithm proposed by Heidari et al.^[9]^ in 2019 that mimics the way Harris eagles find and chase prey in nature, including global exploration, local besiege and pounce behaviour. However, similar to the other SI algorithms, the HHO still has some limitations, such as the multiplicity of solutions generated by a randomized policy that is finite in the initialization phase. Moreover, because global exploration is only performed in the first half of the iteration, it is difficult to balance the global exploration and local exploitation capacities by using the escaping energy of prey, so the algorithm may converge slowly, has low solution accuracy and prematurely falls into a local optimal solution. **In this paper, to conquer the limitations of HHO, a chaotic sequence chaotic sequence recombination mechanism (CSRM) strategy is proposed, which enhances the distribution of the initialized solutions in the search space, and accelerates the convergence rate of HHO, and the generalized opposition-based learning recombination mechanism (OBLRM) is proposed, which can have the opportunity to carry out global search in the later period of iteration to jump out of the local optimum and improve the accuracy of the solution.**

12. The authors must include a section as “Experimental Environment”.

Reply：In section 4, the experimental environment is given. All experiments are carried out on a Windows 10 [operating](javascript:;) [system](javascript:;) with MATLAB R2019a on a PC with Inter(R) core i7-10750H and 16 GB RAM memory.
13. Discussion on figure 4 must be given.

Reply：Fig.4(a) and (b) are unimodal functions which have only one minimum value, however, from Fig.4(c) to Fig.4(f) are multimodal functions which have a lot of local minimum values.
14. How the benchmark functions are chosen?

Reply：23 benchmark functions are shown in reference 6, which are categorised as unimodal, multimodal and fixed dimension multimodal, and they are specifically designed to test optimization algorithms. It has been referenced in the article.

15. Table 1 is too long. Moreover, it is not clear how the results of Table 1 are generated.

Reply：In order to fully verify the performance of the improved algorithm in this paper, the results of 23 benchmark functions optimized by 15 optimization algorithms are given in Table 1.  **Due to the limitation of page scope, it can only be decomposed into several sub-tables**. However, we analyze the results in the table from two perspectives. (1) **Evaluation of exploitation capability (F1-F7),** For all unimodal functions excluding F6, the proposed algorithm [acquire](javascript:;)s the best optimal average values and standard deviations, which indicates that the accuracy and stability of the proposed algorithm are the best among 15 optimization algorithms. **(2) Evaluation of exploration capability (F8-F23),** It can be seen from Table 1 that the proposed algorithm outperforms the other algorithms in most of the multimodal functions F8-F13. For F8, the proposed algorithm is inferior only to the ABC algorithm, but superior to all other algorithms. For F9-F11, although most algorithms can obtain the optimal solutions, the convergence speed of the proposed algorithm is the fastest. For F12, the proposed algorithm is inferior only to SFLA algorithm, but superior to all other algorithms. For F13, the proposed algorithm is inferior only to the SFLA, DEHHO and CHHO algorithms, but superior to all other algorithms. However, when compared to the standard HHO algorithm alone, the proposed algorithm is a winner in all conditions, which indicates that proposed is completely superior to the HHO algorithm. For F14, the proposed algorithm is superior to all other algorithms, and the convergence speed is also the fastest. For F15, the PROPOSED algorithm is inferior to only the BHHO algorithm. For F16, the proposed algorithm is inferior to only the SFLA, ABC and DEHHO algorithms, but superior to all other algorithms. For F17, however, the proposed algorithm is superior to only the HHO and HHHO algorithms, but inferior to all other algorithms. For F18, the proposed algorithm is inferior to only the SFLA algorithm, but superior to all other algorithms. For F19, the proposed algorithm is superior to only the HHO and BHHO algorithms, but inferior to all other algorithms. For F20, the proposed algorithm is inferior to only the WOA and CS algorithms, but superior to all other algorithms. For F21-F23, the proposed algorithm is inferior to only the ABC algorithm and superior to all other algorithms. For F14-F23, the proposed algorithm performs barely satisfactory with respect to all other algorithms; however, it still completely outperforms the standard HHO algorithm. In summary, these results show that proposed can provide superior exploration capability. In addition. the proposed algorithm can obtain optimal values for 11 functions out of 23 functions; while it outperforms the standard HHO algorithm on all of the 23 functions. Therefore, the above results reveal that the chaotic sequence and opposite elite learning mechanism can effectively balance the exploitation and exploration capabilities and improve the performance of the HHO algorithm.

16. Section 5 must be renamed as Conclusions and Future Works

Reply：it has been modified.
17. Applications of the proposed scheme can be discussed in the last section.

Reply：it has been modified.
18. All the figures, tables, equations and references must be cited in the text.

Reply：it has been modified. Fig. 1. Different phases of HHO (Heidari et al.^[9]^). Fig. 2. Optimization process of basic HHO (Heidari et al.^[9]^).

19. Key terms of the equations must be defined.

Reply：it has been modified.
20. Improve the English language

Reply: it has been modified by AJE, and the verification code is E65D-842F-B583-0487-1D9C.

21. Include the following references to improve the reference section:

Reply：it has been modified. The following references have been added to the reference section.
• Robust optimization algorithm for the measurement matrix in compressed sensing. CAAI Transactions on Intelligence Technology. DOI: https://doi.org/10.1049/trit.2018.1011
• Ensemble algorithm using transfer learning for sheep breed classification. DOI: 10.1109/SACI51354.2021.9465609
• Image-denoising algorithm based on improved K-singular value decomposition and atom optimization. CAAI Transactions on Intelligence Technology. DOI: https://doi.org/10.1049/cit2.12044
• IFODPSO-based multi-level image segmentation scheme aided with Masi entropy. Journal of Ambient Intelligence and Humanized Computing. DOI: https://doi.org/10.1007/s12652-020-02506-w
• Adaptive multifactorial particle swarm optimization. CAAI Transactions on Intelligence Technology. DOI: https://doi.org/10.1049/trit.2018.1090
• A boosting-aided adaptive cluster-based undersampling approach for treatment of class imbalance problem. International Journal of Data Warehousing and Mining (IJDWM). DOI: 10.4018/IJDWM.2020070104

**Reviewer #2:**

1. English is the main problem, flow specially in related work is missing.

Reply: It has been modified by AJE, and the verification code is E65D-842F-B583-0487-1D9C.

In the introduction section, firstly, the development and application of optimization algorithm are described, secondly, the research situation of HHO algorithm and many HHO variants are summarized, finally, the shortcomings of HHO algorithm are summarized and the improved methods and contributions of this paper are proposed.  Related work is integrated into the introduction section, and 47 references have been cited in this paper.

2. Methodology is not much clear, how the minimum convergence obtained or how global minimum will help to address this concern.

Reply：In this paper, to conquer the limitations of HHO, a chaotic sequence chaotic sequence recombination mechanism (CSRM) strategy is proposed, which enhances the distribution of the initialized solutions in the search space, and accelerates the convergence rate of HHO, and the generalized opposition-based learning recombination mechanism (OBLRM) is proposed, which can have the opportunity to carry out global search in the later period of iteration to jump out of the local optimum and improve the accuracy of the solution.

3. Fitness function is not clear How E is updated.

Reply：E is updated according to equation 3 in this paper.
4. Results are not compared with the existing models. Even datasets is also limited.

Reply：The improved algorithm has been compared with 14 optimization algorithms on the 23 benchmark functions, the results show that the improved algorithm has obvious advantages, indoor positioning experiment can further prove that the performance of original HHO algorithm has been greatly improved, the dataset is measured by ourselves rather than the public dataset.

5. Novelty is limited

Reply：This is an important comment, HHO is a new swarm intelligence optimization algorithm proposed by Heidari et al.^[9]^ in 2019 that mimics the way Harris eagles find and chase prey in nature, including global exploration, local besiege and pounce behaviour. However, similar to the other SI algorithms, the HHO still has some limitations, such as the multiplicity of solutions generated by a randomized policy that is finite in the initialization phase. Moreover, because global exploration is only performed in the first half of the iteration, it is difficult to balance the global exploration and local exploitation capacities by using the escaping energy of prey, so the algorithm may converge slowly, has low solution accuracy and prematurely falls into a local optimal solution. **In this paper, to conquer the limitations of HHO, a chaotic sequence chaotic sequence recombination mechanism (CSRM) strategy is proposed, which enhances the distribution of the initialized solutions in the search space, and accelerates the convergence rate of HHO, and the generalized opposition-based learning recombination mechanism (OBLRM) is proposed, which can have the opportunity to carry out global search in the later period of iteration to jump out of the local optimum and improve the accuracy of the solution.**
