# Supplementary material for: An improved harris hawks optimization algorithm based on chaotic sequence and opposite elite learning mechanism
Source: PLoS One. 2023 Feb 22;18(2):e0281636. doi: 10.1371/journal.pone.0281636 (PMC9946268; doi:10.1371/journal.pone.0281636)
Supplement: Attachment — Submitted filename: Response to Reviewers.docx [file pone.0281636.s002.docx]

**Dear reviewers,**

These are my responses to each question. Thank you for your advice in your busy schedule, I thought about each question carefully and gave an explanation. Due to my limited research skills, there may be some incorrect answers, please criticize and correct me.

Thanks again! Best wishes to you!

**Response to Reviewer #1:**

1. The authors must add section number to get a clear view.

Reply: I have modified it.

2. In the Introduction section, there must be point-wise contribution and the structure of the paper must be given in the last paragraph of the Introduction.

Reply: I have modified it. The contributions of this paper are as follows: (i) a chaotic sequence chaotic sequence recombination mechanism (CSRM) strategy is proposed, which enhances the distribution of the initialized solutions in the search space, and accelerates the convergence rate of HHO;(ii) the generalized opposition-based learning recombination mechanism (OBLRM) is proposed, which can have the opportunity to carry out global search in the later period of iteration to jump out of the local optimum and improve the accuracy of the solution. The rest of the paper is organized as follows. Section 2 gives a detailed overview of the HHO algorithm. The proposed method is introduced in detail in Section 3. In Section 4, the experimental results are analysed. Finally, the conclusions are presented in Section 5.

3. Please note that the caption style of those figures is wrong in which there are multi-figures.

Reply: I have modified it.

4. More discussions on results are required.

Reply: I have modified it. In this paper, we propose a new HHO variant algorithm based on chaotic mapping and an opposite elite learning mechanisms. The chaotic sequence recombination mechanism is applied at the initial population stage to improve the diversity of the population and enhance the exploration ability of the HHO algorithm. The opposite elite learning recombination mechanism is used at the last stage of each iteration, to effectively maintain the optimal individual and enhance the exploitation ability. On the other hand, this method can overcome the shortcoming at the late iteration in the HHO algorithm: inability to perform global search. In addition, the exploitation and exploration capabilities of the HHO algorithm are balanced. The performance of the HHO-CS-OELM algorithm is verified by comparison with 14 optimization algorithms on 23 benchmark functions and an engineering problem which aims to optimize the weights and thresholds of the BP neural network for indoor positioning. Experimental analyses revealed that the proposed algorithm can obtain optimal values for 11 functions out of 23 functions compared to other state-of-the-art SI algorithms, while it outperforms the standard HHO algorithm on all of the 23 functions, so the HHO-CS-OELM algorithm offers competitive results compared to other state-of-the-art SI algorithms, and verified that the HHO-CS-OELM algorithm has better performance than others. The improved BP neural network optimized by the HHO-CS-OELM algorithm reduced the indoor average positioning error from 7.76 cm to 3.81 cm which is obtained by BP neural network, it also revealed that the indoor positioning accuracy obtained by using the BP neural network with the proposed algorithm is significantly improved, which further verifies the superiority of the HHO-CS-OELM algorithm.

To further improve the performance of the HHO-CS-OELM algorithm, outcomes of other benchmark functions still need to be enhanced, and the exploitation and exploration capabilities of the HHO-CS-OELM algorithm need to be investigated further. Moreover, the HHO-CS-OELM algorithm should be used to solve the system optimal parameters, optimal parameters of neural network model, and so on.

5. What are the practical use-cases of the proposed scheme?

Reply: The proposed scheme in this paper mainly improved the performance of HHO algorithm, and then improved the solution accuracy of HHO algorithm in the engineering application field. The practical use-cases included the solution of indoor positioning, the solution of system optimal parameters, and so on.

6. Improve the English language.

Reply: This comment is crucial to improve the quality of my paper. Thank you very much for your comment, I also try to improve my English language to the best of my ability, but English language is not my mother tongue but my second language, so the English language is still to be further improved. I have improved the English language by AJE, the verification code is E65D-842F-B583-0487-1D9C,

the URL is <https://secure.aje.com/en/certificate/verify>.

7. In the Reference section, C, J, etc. are mentioned with each reference. Please delete them. Replace reference no. 3 with https://doi.org/10.1049/cit2.12040. Also, include 10.4018/IJDWM.2020070104.

Reply: I have delete them, and I replace reference no. 3 with <https://doi.org/10.1049/cit2.12040> and 10.4018/IJDWM.2020070104.
